# Mutational landscape of pan-cancer patients with *PIK3CA* alterations in Chinese population

**DOI:** 10.1186/s12920-022-01297-7

**Published:** 2022-07-01

**Authors:** Qingfeng Huang, Yang Zhou, Bowen Wang, Yi Zhao, Fengxia Zhang, Bowen Ding

**Affiliations:** 1 National Clinical Research Center for Cancer, Tianjin Medical University Cancer Institute and Hospital, West Huan-Hu Road, Ti Yuan Bei, Hexi District, Tianjin, 300060 China; 2 Tianjin’ S Clinical Research Center for Cancer, Tianjin, 300060 China; 3 Key Laboratory of Breast Cancer Prevention and Therapy, Tianjin Medical University, Ministry of Education, Tianjin, 300060 China; 4 Key Laboratory of Molecular Cancer Epidemiology, Tianjin, 300060 China; 5 Genecast (Beijing) Biotechnology Co., Ltd, Beijing, 100000 China; 6 Cheeloo College of Medicine, Shandong University, Jinan, 250000 China

**Keywords:** *PIK3CA* mutation, Chinese population, Concomitant genomic aberrations, Alpelisib

## Abstract

**Purpose:**

To analyze the mutational landscape of pan-cancer patients with *PIK3CA* mutations in Chinese population in real-world.

**Methods:**

We analyzed *PIK3CA* mutation status in sequencing data of cell-free DNA from plasma and genomic DNA from matched peripheral blood lymphocyte in 11,904 Chinese pan-cancer patients, and compared them with genomic data from the Catalogue of Somatic Mutations in Cancer (COSMIC) database. Besides, concomitant genomic aberrations in *PIK3CA*-mutated samples were detected to investigate cancer driver genes, as well as their enriched pathways. Meanwhile, the mutations of Alpelisib targeting genes were screened and their co-alterations were analyzed according to OncoKB definition to identify the potential actionable ones.

**Results:**

The proportion of patients with PIK3CA mutations varied among 21 types of cancer, with the top being BRCA, CESC, SCL, and UCEC. The most common *PIK3CA* mutation hotspots were found to be E545K, E542K and H1047R. The Chinese cohort had significantly lower frequencies of *PIK3CA* mutations in breast and stomach cancers, but markedly higher *PIK3CA* mutation frequencies in large intestine, kidney and lung cancers than the COSMIC cohort. Compared with COSMIC cohort, the mutation frequencies of Alpelisib-targeted genes in breast cancer were significantly reduced in the Chinese cohort. All *PIK3CA*-mutated patients had concomitant genomic aberrations. While the most common concomitant genomic alterations occurred in TP53, EGFR and FAT1, these co-mutated genes were mainly enriched in RTK/RAS pathway, PI3K pathway and P53 pathway. Moreover, 83.6% of patients carrying mutations in Alpelisib-targeted genes had at least one actionable concomitant alteration. Level 1 actionable alteration was identified in LUAD, BRCA, COAD, LUSC, READ, and STAD.

**Conclusion:**

Compared with the Western cohort, the mutation frequency of *PIK3CA* in breast cancer was reduced in the Chinese cohort. RTK/RAS pathway, PI3K pathway and P53 pathway were identified as the most common co-mutation pathways, suggesting that they may potentially serve as targets for possible Alpelisib-based combination therapy.

**Supplementary Information:**

The online version contains supplementary material available at 10.1186/s12920-022-01297-7.

## Introduction

Phosphatidylinositol 3-kinase (PI3K)/protein kinase B (AKT)/mammalian target of rapamycin (mTOR) signaling pathway participates in a variety of cellular and biological processes such as maintenance of protein synthesis, cell proliferation, metastasis, apoptosis, metabolism, and cell cycle regulation, thus playing an important role in tumorigenesis and cancer development [1]. PI3Ks are key components of PI3K/AKT/mTOR signaling pathway, which are categorized into 3 classes according to their structural and biochemical properties [1]. Among them, class I has been most widely studied. It has been shown that Class IA PI3Ks are closely correlated with tumor biological behavior [2]. Class IA PI3Ks comprise a regulatory subunit p85 and a catalytic subunit p110. While the regulatory subunit p85 has three isoforms p110α, p110β and p110δ, p110α is encoded by *PIK3CA* gene that is considered one of the most frequently mutated genes in cancers, particularly in breast cancer [3].

*PIK3CA* is expressed in normal brain, lung, breast, gastrointestinal tract, cervix, ovary, and other tissues. It acts as an oncogene and has many important physiological functions, such as regulating somatic cell proliferation, differentiation, and survival. *PIK3CA* usually exists in an inactive form and its expression is too low to be detected. Mutations in *PIK3CA* may lead to its overexpression. Studies have shown that *PIK3CA* mutations can lead to abnormally enhancement of PI3Ks catalytic activity, and then activate the PI3K/AKT pathway, thereby leading to cell carcinogenesis [4]. It has been reported that *PIK3CA* mutations occur in approximately 30% of human solid tumors, including colorectal cancer, glioblastoma, gastric cancer, breast cancer, and lung cancer, and so forth [1]. The mutation frequencies in breast, colorectal and lung cancers are 7.5–35.5%, 16.9–30.6%, and 0.6–20%, respectively [1]. *PIK3CA* mutations are relatively rare in tumors of the biliary system and diffuse large B-cell lymphoma [5, 6]. It has been demonstrated that *PIK3CA* mutations have prognostic value in patients with hormone receptor positive (HR+)/human epidermal growth factor receptor 2 negative (HER2-) metastatic breast cancer, while becoming an important potential target for systemic therapy [7].

PI3K inhibitors can improve the prognosis of cancer patients through blocking PI3K/AKT/mTOR signaling pathway. Alpelisib is the first PI3K inhibitor approved by the U.S. Food and Drug Administration (FDA) [8]. It selectively inhibits p110α and blocks the PI3K signaling pathway, thereby suppressing the growth of breast cancer cells carrying *PIK3CA* mutations [9]. Importantly, *PIK3CA* genetic testing and the use of Alpelisib for HR + /HER2- breast cancer patients with PIK3CA mutation have been included in the National Comprehensive Cancer Network (NCCN) breast cancer clinical practice guidelines. Moreover, the combination therapy using Aplelisib and Fulvestrant has become the first choice for treatment of postmenopausal HR + /HER2- breast cancer patients with *PIK3CA* mutations [8]. Currently, the blocking of normal pathways beyond the target and Alpelisib-caused off-target effects are still thorny issues [4]. Therefore, it is imperative to delineate the comprehensive landscape of *PIK3CA* mutations across different tumor types and ethnicities. Characterization of somatic mutations for each cancer sample can facilitate identification of the drug targets and subsequent selection of the targeted drugs. Furthermore, identification of co-existing genomic aberrations may help to choose Alpelisib-based combination therapy.

Herein, we analyzed the epidemiologic landscape of *PIK3CA* mutations in a cohort of 11,904 Chinese pan-cancer samples and identified the unique mutation features of *PIK3CA* gene in the Chinese population based on comparative studies between the Chinese and Western cohorts. Meanwhile, we investigated the concomitant genomic alterations of *PIK3CA* mutations, analyzed the tumor driver genes and their enriched pathways, and screened for genes that can be used as drug targets. This study may help to determine the mechanisms of tumor development as well as the possibility of Alpelisib-based combination therapy.

## Materials and methods

### Data sources

Sequencing data on 11,904 solid tumors were obtained from the in-house database that was created based on peripheral blood lymphocyte (PBL) samples and matched circulating cell-free DNA (cfDNA) samples from Chinese cancer patients collected between October 26, 2018 and May 14, 2020. Ninety percent of these patients were the unresectable ones with advanced cancer (Additional file 1: Table S1). The median age was 61. Among 11,904 patients, 6912 were female and 4992 were male. The cancer type distribution among the solid tumors was shown in Additional file 3: Figure S1. Somatic mutation data on the western cohort were collected from the Catalogue of Somatic Mutations in Cancer (COSMIC, May 2019 version) databases (https://cancer.sanger.ac.uk/cosmic). The comparison of somatic mutations between the Chinese and COSMIC cohorts was made. In this study, we comparatively analyzed a total of 14 cancer types between the two cohorts. Cancer classification in the COSMIC cohort was used as a standard for cancer typing. In this case, lung cancer included small cell lung cancer (SCL) and non-small-cell lung carcinoma (NSCL), such as adenocarcinoma (LUAD), squamous cell carcinoma (LUSC), and others, while large intestine cancer represented colon adenocarcinoma (COAD) and rectum adenocarcinoma (READ). All the procedures were carried out in accordance with relevant guidelines and regulations.

### DNA extraction

The method for DNA extraction was as described by Xu et al. [10]. Genomic DNA was extracted from the PBLs using the TGuide S32 Magnetic Blood Genomic DNA Kit (Tiangen, China). Plasma cfDNA was isolated with the MagMAX Cell-Free DNA (cfDNA) Isolation kit (Thermo Fisher Scientific, USA). The concentration of extracted DNAs was measured using the Qubit dsDNA HS Assay Kit (Thermo Fisher Scientific, USA), and the DNA quality was assessed by the Agilent 2100 BioAnalyzer (Agilent, USA).

### Library construction

Library construction based on previous research [10]. The 40–200 ng genomic DNA from PBL was fragmented to a size of approximately 200 base pairs (bp) by an enzymatic method (5 × FEA Enzyme Mix; Qiagen, CN). Obtain the final library according to their method [10]. Meanwhile, cfDNA libraries were prepared with 15–50 ng fragmented DNA extracted from plasma using a VAHTS Universal DNA Library Prep Kit (Vazyme, CN) following the manufacturer’s instructions. The libraries were quantified with AccuGreen High Sensitivity dsDNA Quantitation Kit (Biotium, USA), and the size of libraries was determined by using an Agilent Bioanalyzer 2100 system (Agilent, USA) [10].

### Target area capture and sequencing

Two designed panels used in this study included 534 and 762 genes frequently mutated in solid tumors, which span 1.7 and 1.9 Mb regions in the human genome, respectively. For 11,904 patients involved in this study, 10,005 patients were captured using the panel with 534 genes, and 1899 patients captured using the panel with 762 genes. Both panels covered all exons of PIK3CA, thus the patients captured by the two panels could be put together for statistical analysis in this study. Next, the capture of the targeted regions, hybridization reactions and subsequent washes were performed according to the previous method of the members of our research group, and the captured libraries were sequenced [10]. The average sequencing depth of PBL Genomic DNA and plasma cfDNA was 805 × and 7338 × , respectively.

### SNV/INDEL detection

SNV/INDEL detection were performed as previously described [10]. The sequenced reads were trimmed with Trimmomatic (v0.36) [11] to remove adaptor sequences and low-quality bases. Afterwards, clean reads were mapped to the human reference genome (hg19) using BWA (v0.7.17) [12], sorted and masked for duplications using Picard (v2.23.0) [13]. SNVs and InDels were identified using VarDict (v1.5.1) [14] in each PBL and cfDNA sample, and complex mutations were called with FreeBayes (v1.2.0) [15]. To exclude false-positive SNVs and InDels, those appearing in the blacklist including sequence-specific errors, repeat regions, segmental duplications, and lowly mappable regions recorded in ENCODE were removed [16]. The mutations were filtered and annotated using ANNOVAR (2015Jun17) [17], and synonymous mutations were not considered in this study.

### Screening of somatic and germline mutations

In this study, we defined mutations detected only in cfDNA and those present in both PBL and cfDNA with a sequencing depth of at least 30 × and a VAF of no less than 20% as candidate somatic mutations and germline mutations, respectively. Somatic mutations were retained if they met the following criteria: (1) sequencing was performed with a sequencing depth of no smaller than 100 × ; (2) the number of reads supporting the variant allele was no less than 2; (3) the VAF of candidate mutations was no less than 0.3%; and (4) the minor allele frequency (MAF) of somatic mutations in databases gnomAD [18] and ExAC [19, 20] was no greater than 0.2%. Moreover, somatic mutations recorded in dbSNP [21] database but not in COSMIC [22, 23] database and those in the HLA loci were filtered out. In the meantime, pathogenic germline mutations in ClinVar database were retained, including “Pathogenic” and “Likely pathogenic” ones [24]. Besides, we retained mutations with a MAF of less than 2% in databases gnomAD and ExAC even though they were not recorded in ClinVar database.

### CNV detection

After correcting for GC content, target region length, and read counts, the copy number and gene specificity score (GCS) were calculated by using 30 normal blood samples as a control. GCS represents the degree of difference in the gene level between the test sample and control. Copy number variation (CNV) was determined by joint statistical significance test on GCS and absolute value of the copy number.

### Statistical analysis

The data were analyzed by using R 3.6.1. Fisher’s exact test was performed to compare data between the Chinese and COMSIC cohorts. *P* values < 0.05 were considered statistically significant.

## Results

### Characterization of PIK3CA mutations in the Chinese cohort

**Fig. 1:**
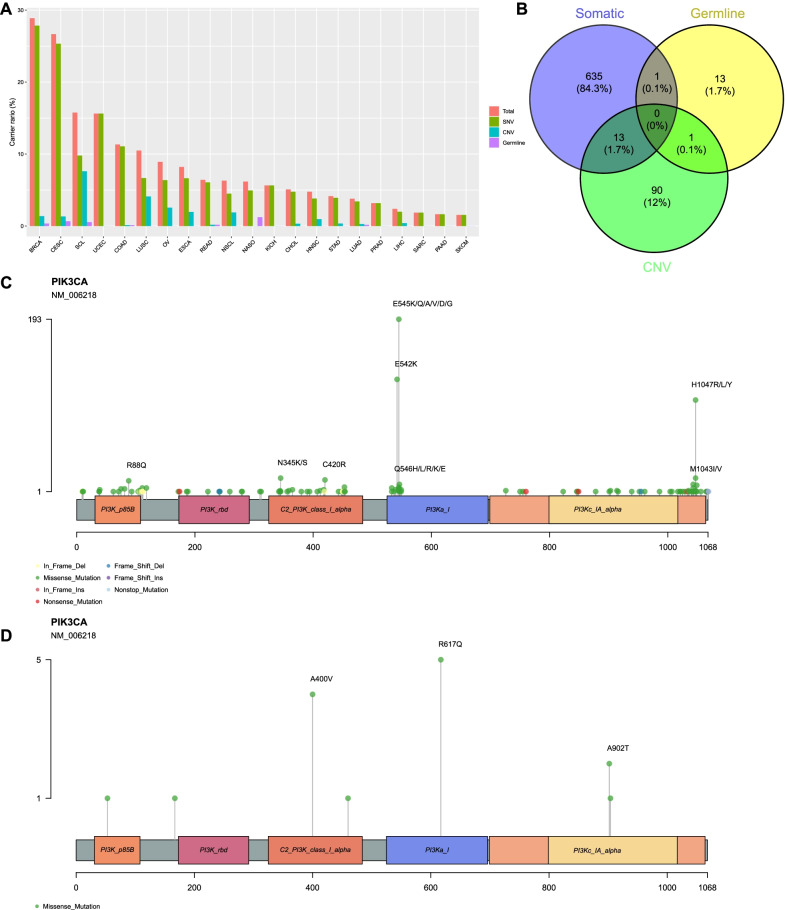
Characterization of *PIK3CA* mutations in Chinese cohort. **A** Frequencies of *PIK3CA* mutations in different cancer types. **B** Distribution of *PIK3CA* mutation types. **C** Schematic representation of *PIK3CA* functional domains and somatic mutation analysis. **D** Schematic representation of *PIK3CA* functional domains and germline mutation analysis

753 out of 11,904 patients (6.33%) had *PIK3CA* mutations, with the highest mutation frequencies being detected in breast invasive carcinoma (BRCA), cervical squamous cell carcinoma and endocervical adenocarcinoma (CESC), SCL, and uterine corpus endometrial carcinoma (UCEC) (Fig. 1A). Further analysis showed that among 753 patients with *PIK3CA* mutations, 649 harbored somatic mutations that comprised 144 mutation types and mainly involved hotspots such as E545K/Q/A/V/D/G, E542K, H1047R/ L/Y, and so forth. Meanwhile, 15 out of 753 patients with *PIK3CA* mutations were found to carry germline mutations that contained 7 mutation types and mainly involved hotspots including R617Q, A400V, and A902T. In addition, the CNV in *PIK3CA* with an increased copy number was identified in 104 patients (Fig. 1B–D). Statistical analyses on the frequencies of mutation hotspots and CNVs in *PIK3CA* among different cancers types revealed that the CNV was most commonly detected in SCL and LUSC, while E545K, E542K and H1047R were the most frequently identified mutations in most cancers (Additional file 4: Figure S2).

### Comparison of the mutational landscapes between the Chinese and COSMIC cohorts

**Fig. 2:**
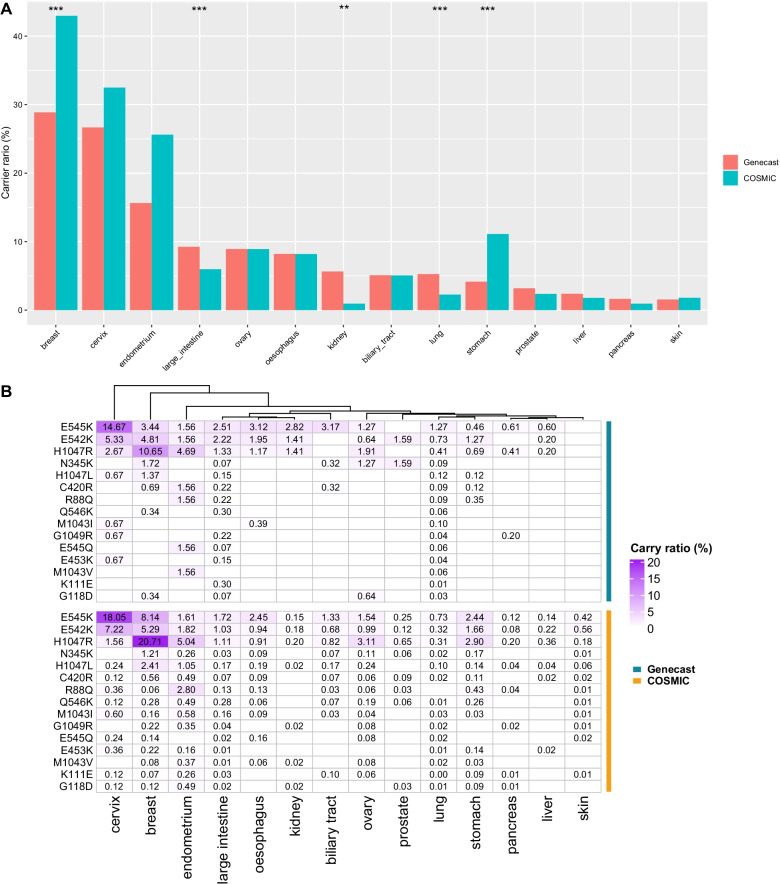
Mutational landscape comparison between Chinese and COSMIC cohorts. **A** Comparison of *PIK3CA* mutation frequencies in different cancer types between Chinese and COSMIC cohorts. **B** Frequencies of top 15 *PIK3CA* mutation hotspots in different cancers types between Chinese and COSMIC cohorts. *P < 0.05, **P < 0.01, ***P < 0.001

We compared the *PIK3CA* mutation frequencies in different cancer types between the Chinese cohort and COSMIC database. As shown in Fig. 2A, *PIK3CA* mutation frequencies of large intestine, kidney and lung cancers in the Chinese cohort were significantly higher than those in the COSMIC cohort, while the mutation frequencies of breast and stomach cancers in the Chinese cohort were notably lower than the COSMIC cohort. Moreover, compared with the COSMIC cohort, *PIK3CA* mutations in most cancer types except for large intestine cancer and lung cancer were more concentrated at hotspots mainly involving E545K, E542K and H1047R in the Chinese cohort (Fig. 2B).

### Concomitant genomic alterations in patients with PIK3CA mutations

**Fig. 3:**
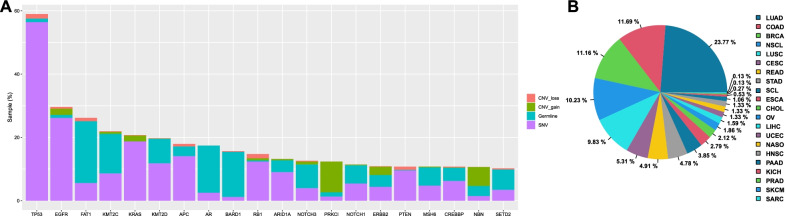
Concomitant genomic alterations in patients with *PIK3CA* mutations. **A** Frequencies of the top 20 frequent co-aberrations of *PIK3CA*. **B** Distribution of co-aberrations of *PIK3CA* in different cancers types

We next analyzed concomitant genomic alterations in all tumor samples carrying *PIK3CA* mutations. As depicted in Fig. 3A, the most common concomitant genomic alterations involved *TP53*, *EGFR* and *FAT1*. Notably, LUAD was identified as the cancer type with the highest frequency of co-mutations, followed by COAD, BRCA, and NSCL (Fig. 3B). Based on a large-cohort-study [25], we selected the driver genes in different cancers and examined concomitant aberrations on these genes in the tumor samples. As illustrated in Additional file 5: Figure S3, the most common co-occurring driver gene in large intestine cancer was *KRAS* (67%), followed by *APC* (62%) and *TP53* (62%). Meanwhile, *TP53* (54%) and *ERBB2* (19%), *EGFR* (70%) and *TP53* (61%), *TP53* (75%) and *CDKN2A* (32%) were identified as the most common co-occurring driver genes in BRCA, LUAD, and LUSC, respectively.

### Pathway analysis of concomitant alterations

**Fig. 4:**
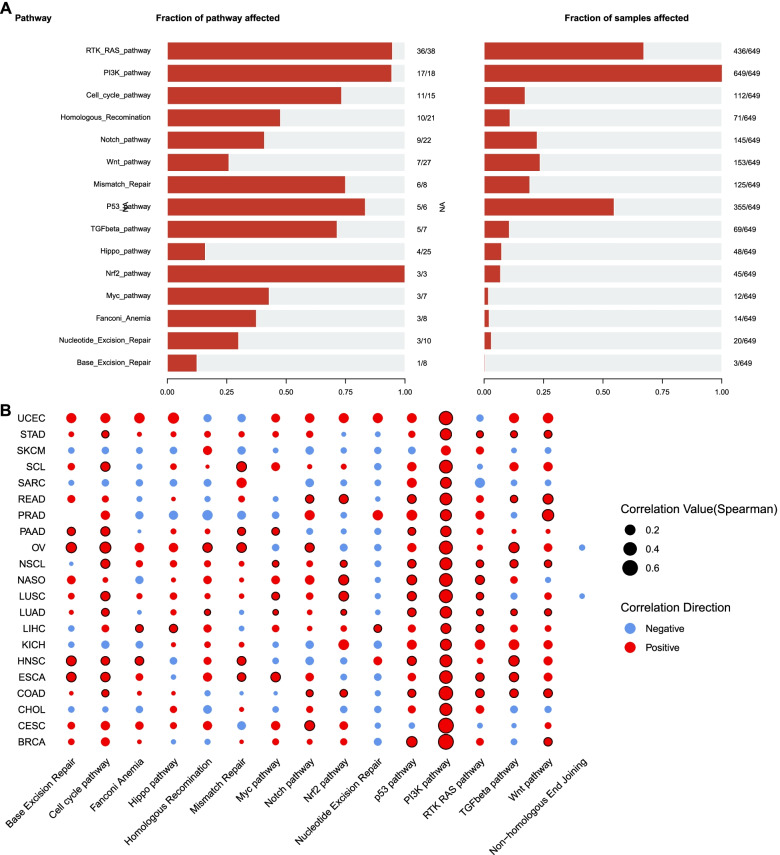
Pathway analysis of concomitant alterations in patients with *PIK3CA* mutations. **A** Frequencies of co-mutated genes and samples with mutations in 18 pathways. **B** The correlation between the *PIK3CA* mutations and the mutations of these 18 pathway-related genes in different cancers

We further analyzed enrichment of the concomitant mutated genes in 18 pathways, including 10 oncogenic pathways and 8 repair-related pathways [26, 27]. As shown in Fig. 4A, in all cancer patients with *PIK3CA* mutations, the concomitant mutated genes were mainly enriched in the RTK/RAS pathway, PI3K pathway and P53 pathway, which was similar to the results analyzed in BRCA, LUAD and LUSC, while the concomitant mutated genes also significantly enriched in the Wnt pathway in large intestine cancer (Additional file 6: Figure S4). Moreover, correlation analysis revealed that in most cancers, *PIK3CA* mutations were positively correlated with the cell cycle pathway, RTK/RAS pathway, PI3K pathway, P53 pathway, and Wnt pathway, while being negatively correlated with nucleotide excision repair-related pathways (Fig. 4B).

### Characterization of mutations in Alpelisib-targeted genes in the Chinese and COSMIC cohorts

**Fig. 5:**
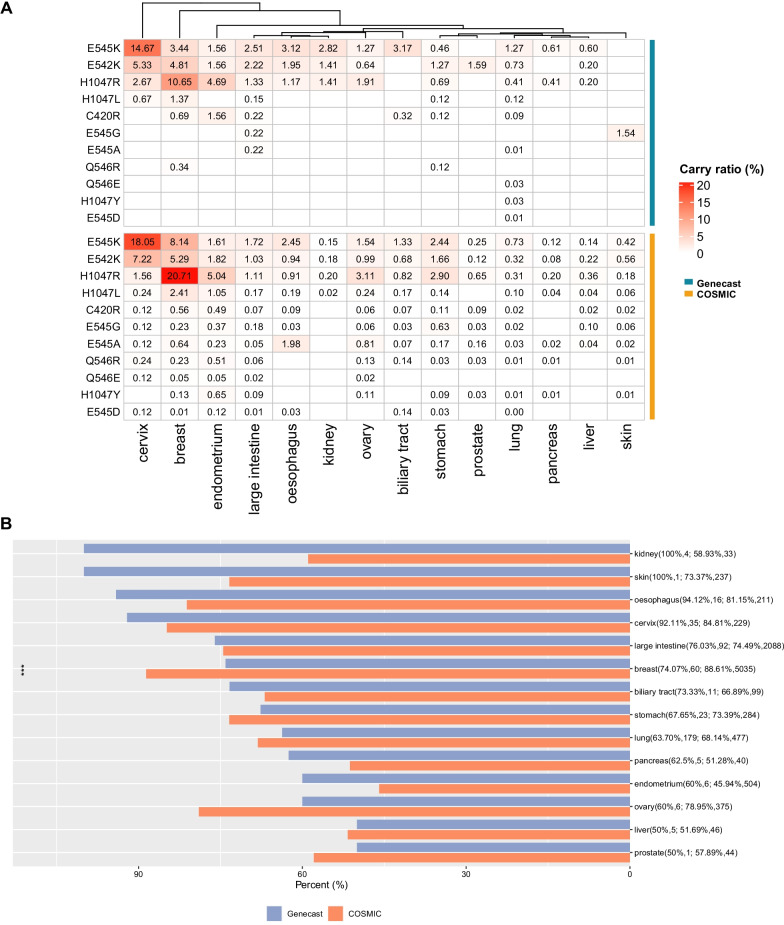
Characterization of Alpelisib target mutations in Chinese and COSMIC cohorts. **A** Mutation frequencies of 11 Alpelisib targets in different cancer types in the Chinese and COSMIC cohorts. **B** Comparison of frequencies of total Alpelisib target mutations in different cancers types between Chinese and COSMIC cohorts. ***P < 0.001

We compared therapeutic benefits of Alpelisib between the Chinese and COSMIC cohorts by analyzing mutations in Alpelisib-targeted genes (Additional file 2: Table S2). For most cancers, E545K, E542K and H1047R were identified as the main mutations of Alpelisib-targeted genes in the Chinese cohort, while mutations of those genes were dispersedly distributed in the COSMIC cohort (Fig. 5A). In addition, we observed that compared with COSMIC cohort, the mutation frequency of Alpelisib-targeted genes in breast cancer was reduced in the Chinese cohort (Fig. 5B).

### Identification of concomitant actionable mutations in patients with mutations in Alpelisib-targeted genes

**Fig. 6:**
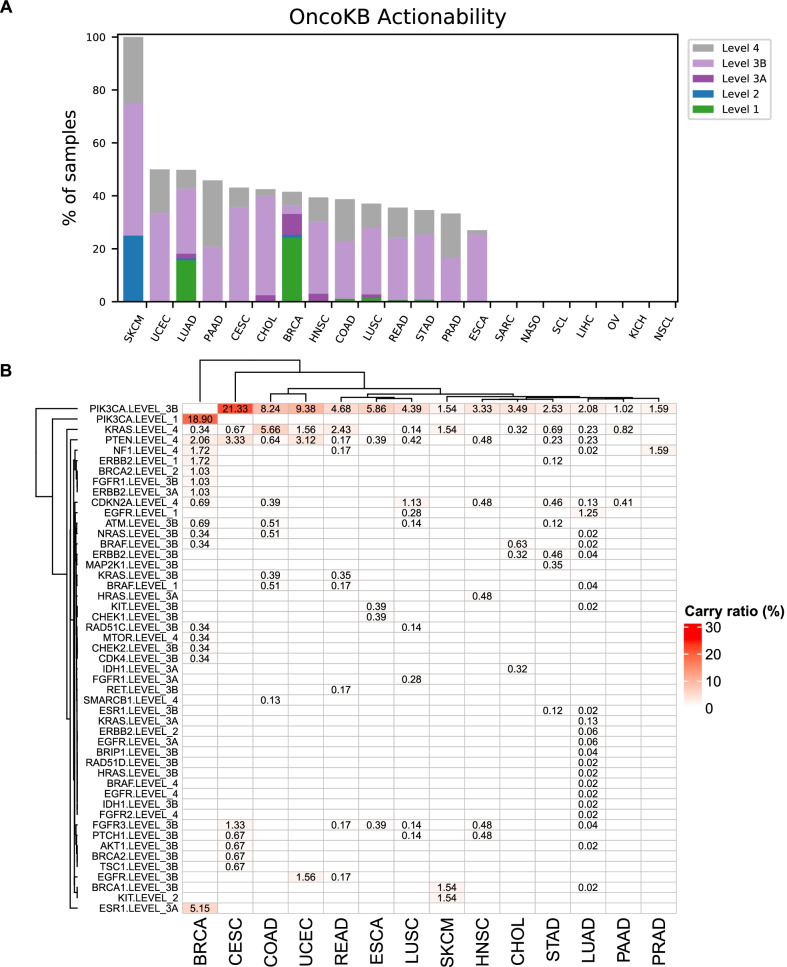
Actionable mutations concomitant with Alpelisib target mutations. **A** Frequencies of patients harbored actionable co-alterations as defined by OncoKB. **B** Potential actionable alterations in Alpelisib target co-alterations defined as OncoKB level 1 to 4 in different cancer types

We screened for candidate actionable co-alterations by using the OncoKB classification [28]. In this study, 83.6% of patients carrying mutations of Alpelisib-targeted genes were found to harbor at least one actionable co-alteration. Strikingly, level 1 actionable alteration was identified in LUAD, BRCA, COAD, LUSC, READ, and STAD (Fig. 6A). In these cases, actionable mutations on *EGFR* (1.25%) or *BRAF* (0.04%), FDA-approved biomarkers for target therapies (level 1), were detected in LUAD cases. Meanwhile, *ERBB2* mutations (1.72%) were identified as level 1 actionable co-alterations in BRCA cases (Fig. 6B).

## Discussion

To the best of our knowledge, this is the first study to characterize genomic and epidemiological landscapes of *PIK3CA* mutations in cfDNA of Chinese pan-cancers population. In this study, we found that in the Chinese cohort, *PIK3CA* mutations mainly occurred in BRCA, CESC, SCL, and UCEC, while most of the mutations were clustered within the helical domain (E542K and E545K) or the kinase domain (H1047R), providing consistent data with the previous studies [1, 29]. We further compared *PIK3CA* mutations between the Chinese and Western cohorts. The comparative studies revealed that the Chinese cohort had a significantly higher frequency of *PIK3CA* mutations in large intestine, kidney, and lung cancers, but a markedly lower mutation frequency in breast and stomach cancers than the COSMIC cohort. Notably, compared with the COSMIC cohort, *PIK3CA* mutations in most cancer types except for large intestine cancer and lung cancer were more concentrated at hotspots in the Chinese cohort.

The present study identified concomitant genomic alterations in all tumor samples carrying *PIK3CA* mutations. Here, we showed that the most common concomitant genomic alterations involved *TP53*, *EGFR* and *FAT1*, while the co-mutated genes were mainly enriched in RTK/RAS pathway, PI3K pathway and P53 pathway. While RTK/RAS and PI3K pathways have been shown to be involved in cell proliferation, differentiation and survival, aberrant activation of these pathways can induce cancer cell growth and metastasis [30]. It has been demonstrated that p110α could complex with activated RTKs, possibly mediating growth factor signaling [1]. P53 activation can also induce cell cycle arrest, senescence and apoptosis. Herein, we found an elevated mutation frequency of Wnt pathway in large intestine cancer, showing consistent data with the study of Lee et al. [31]. The study has shown that excessive activation of Wnt pathway, mainly caused by the inactivation of the *APC* gene, can lead to the occurrence of CRC [31]. The above findings provided some potential pathways that might be associated with PI3K specific inhibitor resistance, which might facilitate future screening of drugs that could be used in combination with PI3K specific inhibitors.

PI3K plays an important role in human cancers, thereby becoming an attractive therapeutic target. The development of isoform-selective PI3K inhibitors and co-administration of these inhibitors with other therapeutic agents have greatly improved the therapeutic effect of cancers. Mayer et al. [32] showed that p110α-specific inhibitor Alpelisib combined with letrozole was safe and effective in the treatment of patients with resistant ER + metastatic breast cancer, while Alpelisib displayed a more obvious effect on breast cancer patients carrying *PIK3CA* mutations. Juric and André et al. [33, 34] found that Alpelisib-fulvestrant combination therapy had a significantly better effect on HR + /HER2- breast cancer patients with *PIK3CA* mutations than fulvestrant alone. The NCCN breast cancer clinical practice guidelines recommended that Alpelisib plus fulvestrant combination therapy should be used as the first choice for the treatment of HR + /HER2- breast cancer patients carrying *PIK3CA* mutations. The present study found that while the mutation frequency of Alpelisib-targeted genes in breast cancer in the Chinese cohort was lower than that in COSMIC cohort, there were no significant differences in the mutation frequencies in other cancer types between the two cohorts, indicating that breast cancer patients in Western population could benefit more from Alpelisib than those in Chinese population. We further screened for concomitant actionable mutations in cancer patients carrying mutations in Alpelisib-targeted genes. Notably, actionable mutations on *BRAF*, FDA-approved biomarkers for target therapies, were identified in LUAD, COAD and READ. At present, small molecule inhibitors of BRAF p.V600E, such as Sorafenib and PLX4720, have been developed as therapeutic agents for targeting RTK/RAS and PI3K pathways [35]. In this study, we identified ERBB2 mutations as level 1 actionable alterations in BRCA and STAD. A study on combination therapy targeting activated ERBB2 and PIK3CA mutations in cervical cancer found that the majority of cervical tumors could benefit from existing ERBB2/PIK3CA/AKT/mTOR-targeted drugs [36]. All these findings led us to propose that identification of more potentially druggable targets in patients with *PIK3CA* mutations is of great significance for improving the efficacy of Alpelisib.

The limitation of this study is the comparison between cfDNA of Chinese cohort and COSMIC database. Since no corresponding data of liquid biopsy was found in the western pan-cancer cohorts, as an alternative, we selected data from COSMIC database, which was derived from surgically resected tissues, to investigate the similarities and differences in the integrated landscape of pan-cancer patients with *PIK3CA* mutations in Chinese and western population. Liquid biopsies are less sensitive than tissue biopsies which could probably affect the accuracy of comparison results between two cohorts. However, it could better capture the heterogeneity of inter-or intra-tumors and provide the potential of treatment strategy to cancer patients whose tissue samples are not available.

In summary, this study characterized the genomic landscape of *PIK3CA* mutations in various cancers in a large Chinese population and analyzed concomitant genomic alterations/pathways in different cancer types. Moreover, we identified concomitant actionable mutations in cancer patients carrying mutations in Alpelisib-targeted genes. The findings of this study provide valuable input for further research and targeted therapies for *PIK3CA* mutation-carrying cancer.

## Supplementary Information

**Additional file 1: Table S1**. Clinical information.

**Additional file 2: Table S2**. Target of Alpelisib.

**Additional file 3: Figure S1**. Distribution of cancer types in 11904 tumor samples.

**Additional file 4: Figure S2**. Frequencies of PIK3CA mutation hotspots and CNVs in different cancers types.

**Additional file 5: Figure S3**. Driver gene analysis in (A) large intestine cancer (COAD+READ), (B) breast invasive carcinoma (BRCA), (C) lung adenocarcinoma (LUAD) and (D) lung squamous cell carcinoma (LUSC).

**Additional file 6: Figure S4**. Pathway analysis of PIK3CA co-aberrations in (A) large intestine cancer (COAD+READ), (B) breast invasive carcinoma (BRCA), (C) lung adenocarcinoma (LUAD) and (D) lung squamous cell carcinoma (LUSC).

## Data Availability

Data of Western cohort was obtained from the COSMIC databases (https://cancer.sanger.ac.uk/cosmic). The datasets of Chinese cohort can be found in online repositories. The names of the repository/repositories and accession number(s) can be found below: https://ngdc.cncb.ac.cn/gvm/getProjectDetail?project=GVM000313, GVM000313.

## Abbreviations

PI3K: Phosphatidylinositol 3-kinase
AKT: Protein kinase B
mTOR: Mammalian target of rapamycin
HR +: Hormone receptor positive
HER2-: Human epidermal growth factor receptor 2 negative
FDA: U.S. Food and Drug Administration
NCCN: National Comprehensive Cancer Network
PBL: Peripheral blood lymphocyte
cfDNA: Cell-free DNA
COSMIC: The Catalogue of Somatic Mutations in Cancer
GCS: Gene specificity score
CNV: Copy number variations
NSCL: Non-small-cell lung carcinoma
LUAD: Lung adenocarcinoma
LUSC: Lung squamous cell carcinoma
SCL: Small cell lung cancer
COAD: Colon adenocarcinoma
READ: Rectum adenocarcinoma
BRCA: Breast invasive carcinoma
CESC: Cervical squamous cell carcinoma and endocervical adenocarcinoma
UCEC: Uterine corpus endometrial carcinoma
