# Supplementary material for: Mutational landscape of pan-cancer patients with PIK3CA alterations in Chinese population
Source: BMC Med Genomics. 2022 Jul 1;15:146. doi: 10.1186/s12920-022-01297-7 (PMC9248192; doi:10.1186/s12920-022-01297-7)
Supplement: Supplementary file 1 — Additional file 1: Table S1. Clinical information. [file 12920_2022_1297_MOESM1_ESM.docx]

Table S1 Clinical information

|  | **Overlall**  **(N=11904)** |
| --- | --- |
| **Sex** |  |
| female | 6912(58.1%) |
| male | 4992(41.9%) |
| **age** |  |
| Mean (SD) | 60.0 (11.8) |
| Median [Min, Max] | 61 [3.00, 96.0] |
| **stage** |  |
| I | 79 (0.7%) |
| II | 98 (0.8%) |
| III | 1011 (8.5%) |
| IV | 10716 (90.0%) |
