# Supplementary material for: Mutational landscape of pan-cancer patients with PIK3CA alterations in Chinese population
Source: BMC Med Genomics. 2022 Jul 1;15:146. doi: 10.1186/s12920-022-01297-7 (PMC9248192; doi:10.1186/s12920-022-01297-7)
Supplement: Supplementary file 2 — Additional file 2: Table S2. Target of Alpelisib. [file 12920_2022_1297_MOESM2_ESM.docx]

Table S2 Target of Alpelisib

| **Exon** | **Mutation** |
| --- | --- |
| exon7 | C420R |
| exon9 | E542K |
| exon9 | E545A |
| exon9 | E545D[1635G>T only] |
| exon9 | E545G |
| exon9 | E545K |
| exon9 | Q546E |
| exon9 | Q546R |
| exon20 | H1047L |
| exon20 | H1047R |
| exon20 | H1047Y |
