# Supplementary figures and images for: Mutational landscape of pan-cancer patients with PIK3CA alterations in Chinese population
Source: BMC Med Genomics. 2022 Jul 1;15:146. doi: 10.1186/s12920-022-01297-7 (PMC9248192; doi:10.1186/s12920-022-01297-7)

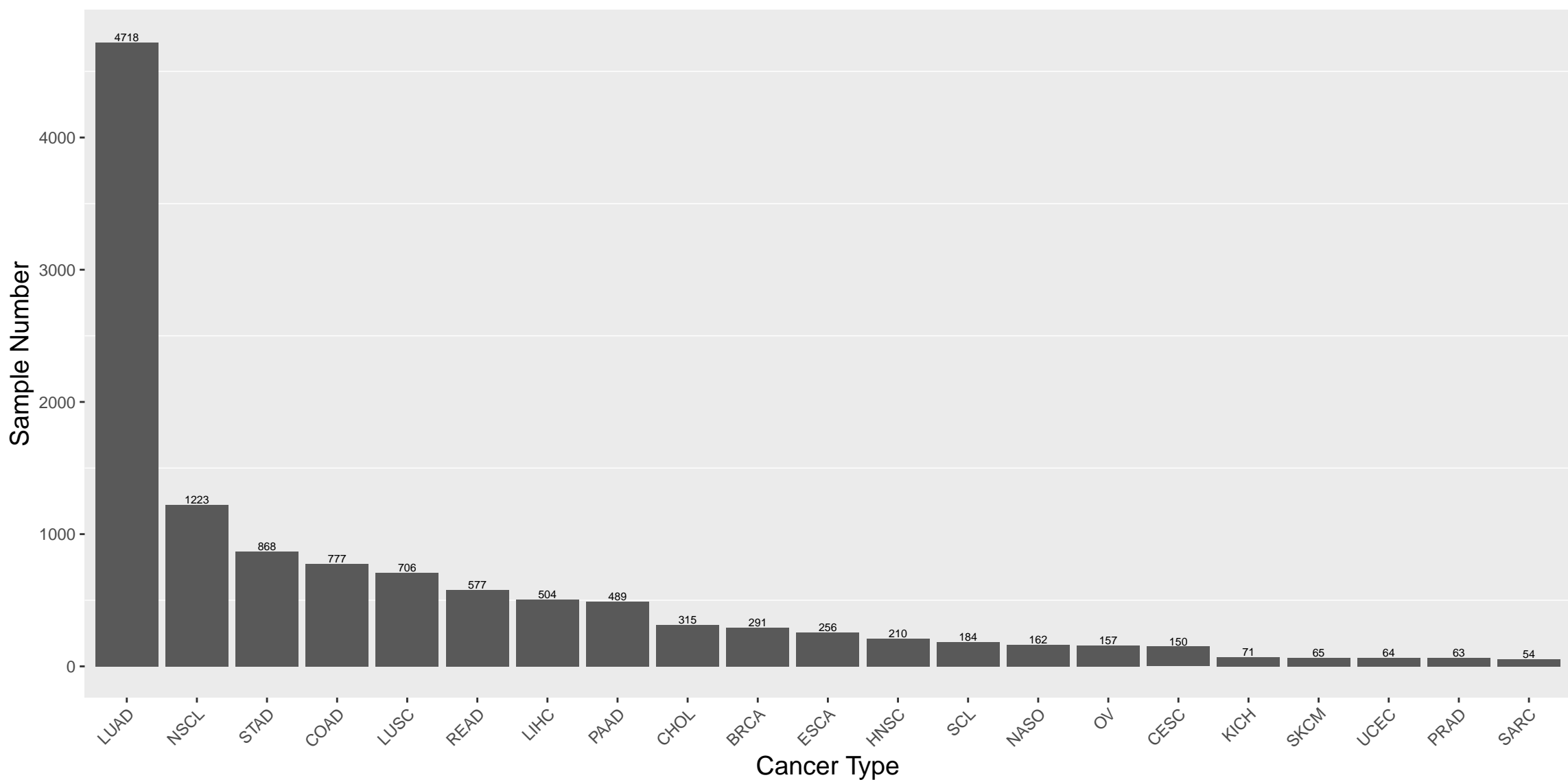

Supplement: Supplementary file 3 — Additional file 3: Figure S1. Distribution of cancer types in 11904 tumor samples. [file 12920_2022_1297_MOESM3_ESM.pdf]

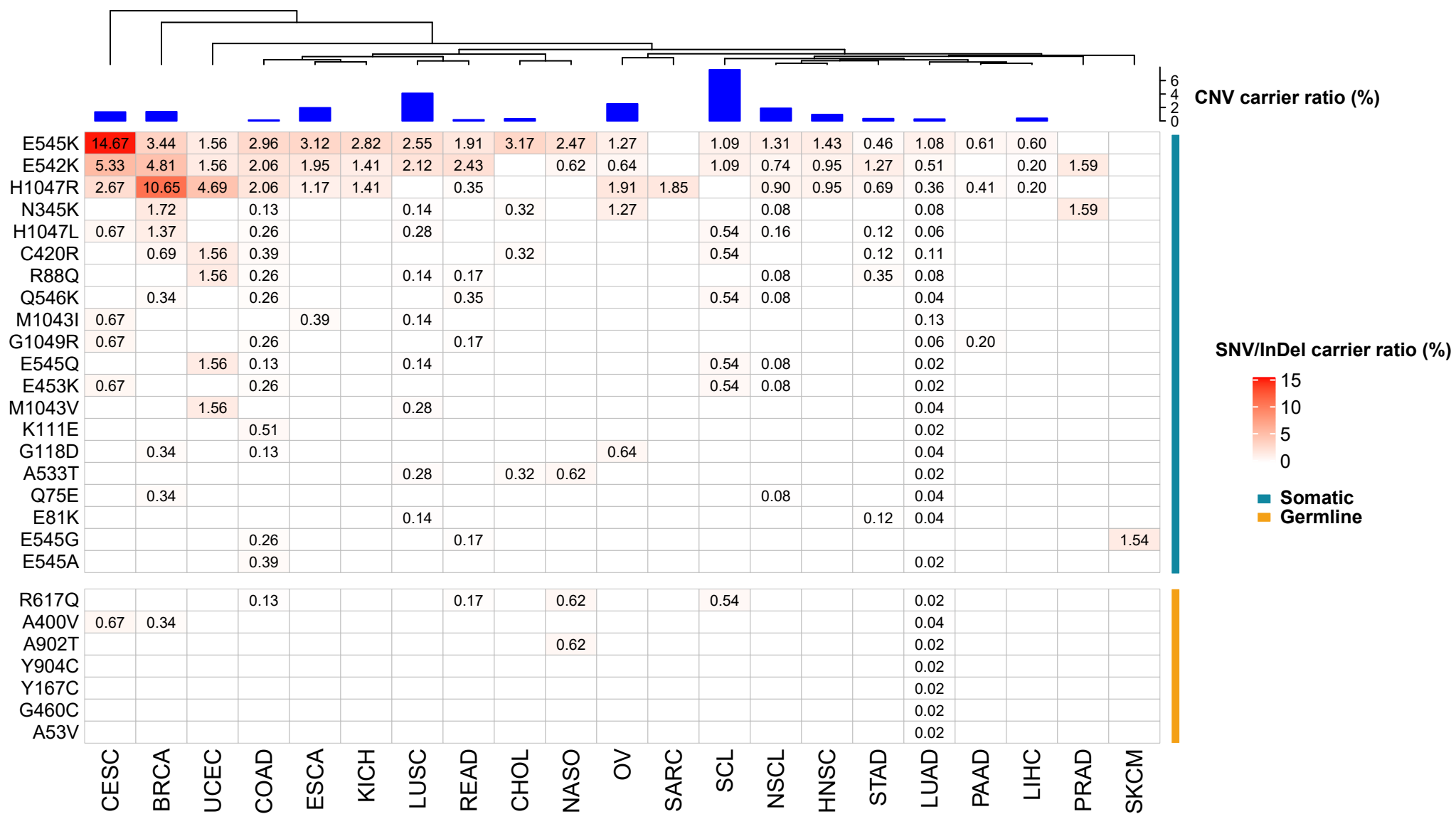

Supplement: Supplementary file 4 — Additional file 4: Figure S2. Frequencies of PIK3CA mutation hotspots and CNVs in different cancers types. [file 12920_2022_1297_MOESM4_ESM.pdf]

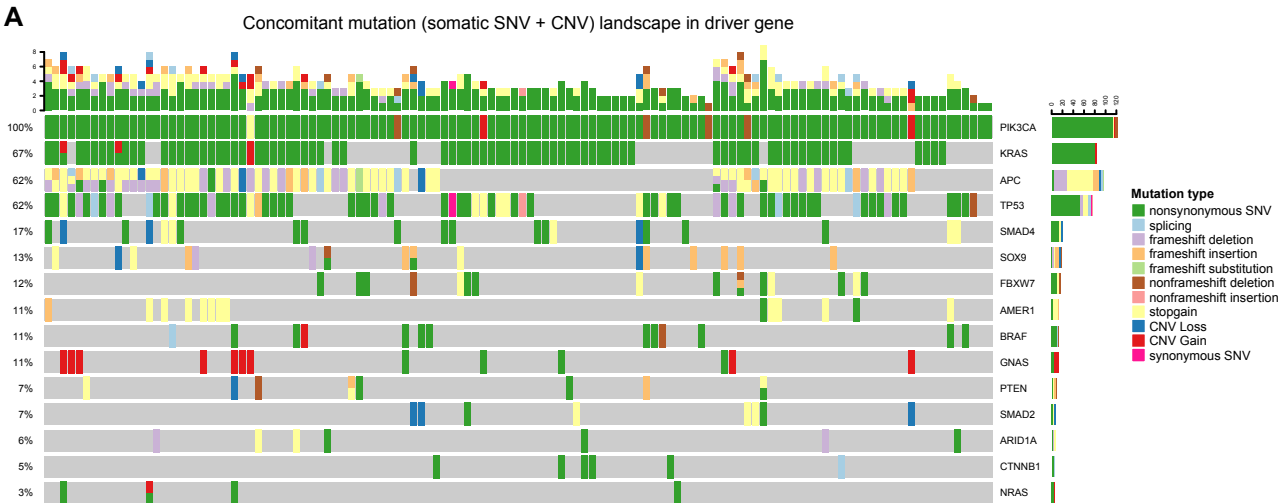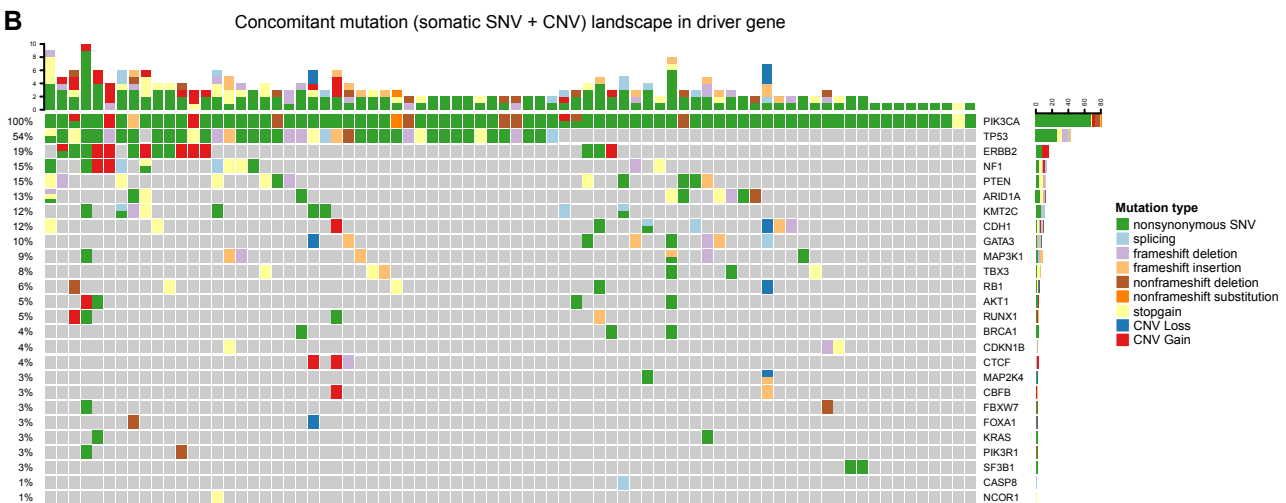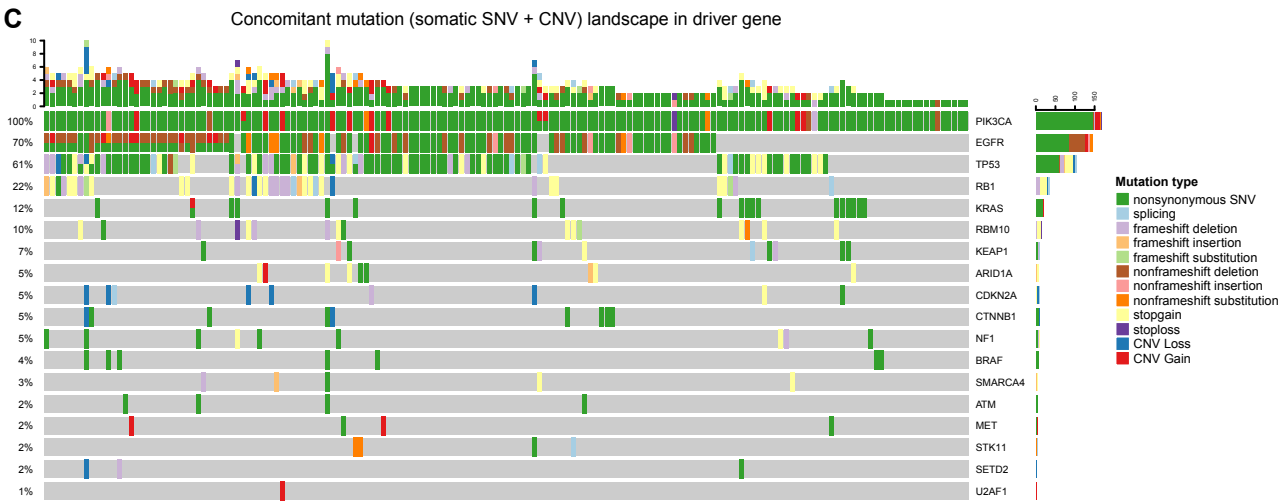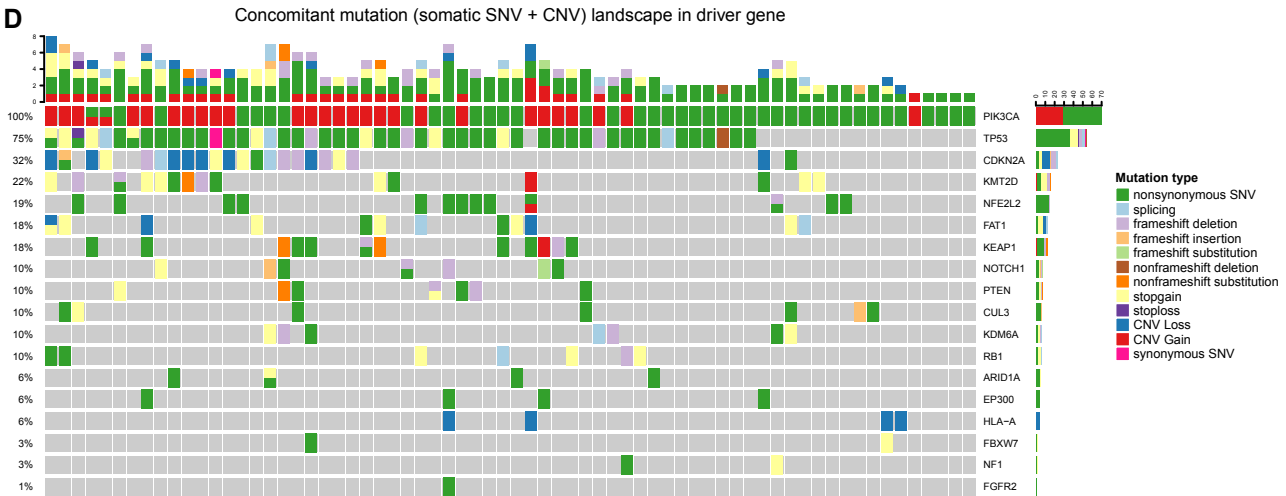

Supplement: Supplementary file 5 — Additional file 5: Figure S3. Driver gene analysis in (A) large intestine cancer (COAD+READ), (B) breast invasive carcinoma (BRCA), (C) lung adenocarcinoma (LUAD) and (D) lung squamous cell carcinoma (LUSC). [file 12920_2022_1297_MOESM5_ESM.pdf]

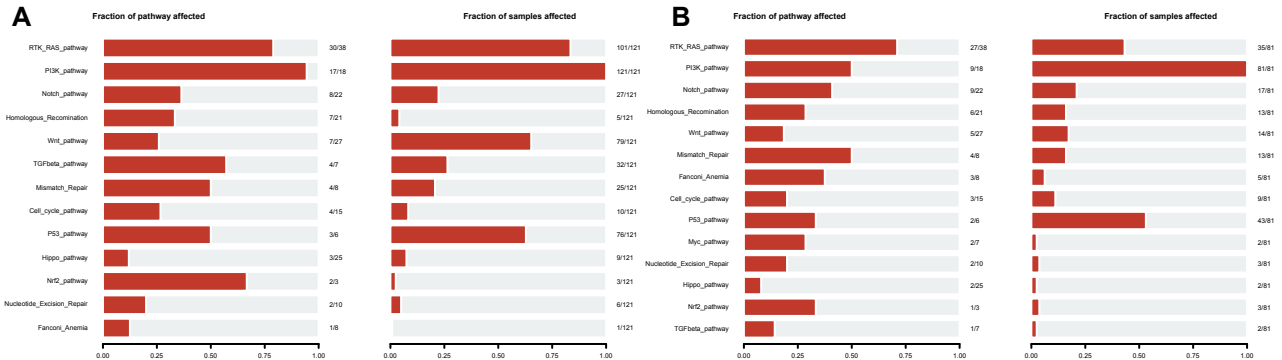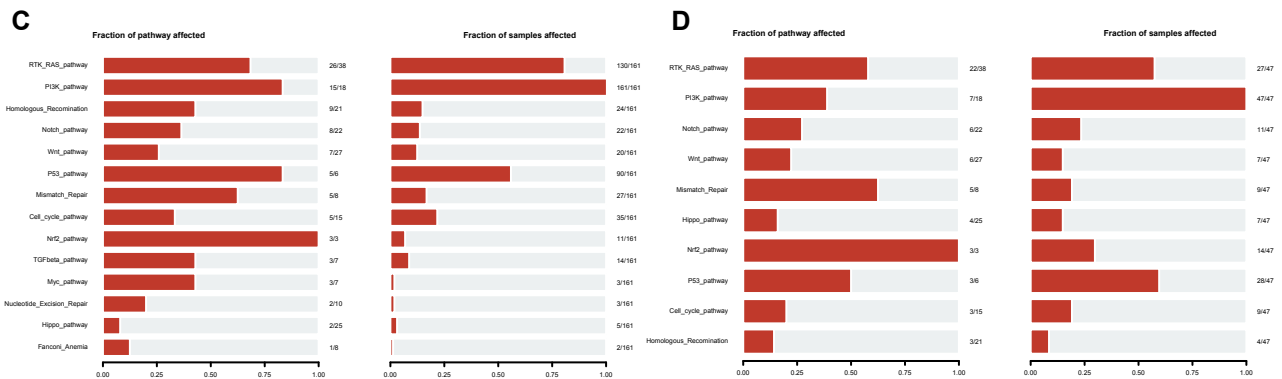

Supplement: Supplementary file 6 — Additional file 6: Figure S4. Pathway analysis of PIK3CA co-aberrations in (A) large intestine cancer (COAD+READ), (B) breast invasive carcinoma (BRCA), (C) lung adenocarcinoma (LUAD) and (D) lung squamous cell carcinoma (LUSC). [file 12920_2022_1297_MOESM6_ESM.pdf]
